# Influence of Botulinumtoxin A on the Expression of Adult MyHC Isoforms in the Masticatory Muscles in Dystrophin-Deficient Mice (Mdx-Mice)

**DOI:** 10.1155/2016/7063093

**Published:** 2016-08-07

**Authors:** Ute Ulrike Botzenhart, Constantin Wegenstein, Teodor Todorov, Christiane Kunert-Keil

**Affiliations:** Department of Orthodontics, Carl Gustav Carus Campus, TU Dresden, Fetscherstrasse 74, 01307 Dresden, Germany

## Abstract

The most widespread animal model to investigate Duchenne muscular dystrophy is the mdx-mouse. In contrast to humans, phases of muscle degeneration are replaced by regeneration processes; hence there is only a restricted time slot for research. The aim of the study was to investigate if an intramuscular injection of BTX-A is able to break down muscle regeneration and has direct implications on the gene expression of myosin heavy chains in the corresponding treated and untreated muscles. Therefore, paralysis of the right masseter muscle was induced in adult healthy and dystrophic mice by a specific intramuscular injection of BTX-A. After 21 days the mRNA expression and protein content of MyHC isoforms of the right and left masseter, temporal, and the tongue muscle were determined using quantitative RT-PCR and Western blot technique. MyHC-IIa and MyHC-I-mRNA expression significantly increased in the paralyzed masseter muscle of control-mice, whereas MyHC-IIb and MyHC-IIx/d-mRNA were decreased. In dystrophic muscles no effect of BTX-A could be detected at the level of MyHC. This study suggests that BTX-A injection is a suitable method to simulate DMD-pathogenesis in healthy mice but further investigations are necessary to fully analyse the BTX-A effect and to generate sustained muscular atrophy in mdx-mice.

## 1. Introduction

The stomatognathic system is based on a close mutual and functional network of different hard and soft tissues from those the masticatory muscles illustrate as an essential component. They are one of the strongest muscles of the body [1].

In mammalians, muscle contraction is possible due to highly organized motor units consisting of a motor neurone located in the brain stem [2], its axons, and a colony of corresponding fibres [2, 3]. Motor units show a large variability in morphological and physiological characteristics [2] and can be distinguished on the basis of the differences in contraction time, twitch tension, susceptibility to fatigue, and histochemical staining [3]. Three classes of motor units called slow fatigue resistant, fast fatigable, and fast fatigue resistant were initially identified in mammalian skeletal muscles composed by slow oxidative type I and fast glycolytic type IIa and IIb fibres, respectively [3–5]. A fourth motor unit type with fast contractile characteristics and intermediate fatigability composed by type IIx fibres was subsequently detected in rat skeletal muscles [6, 7]. Due to that fact, the most informative methods to delineate muscle fibre types are based on specific myosin profiles, especially the myosin heavy chain isoform complement [8] possibly being more related to functional behaviour of jaw muscle motor units than past histochemical classifications [6].

Myosin heavy chains exist in multiple isoforms that are differentially distributed in the various fibre types [9]. At least, four different isoforms of myosin heavy chains are expressed in adult skeletal muscle: slow isoform type I coded by Myh7 gene [10] and fast isoforms type IIa, IIx/d, and IIb [8, 11–14] coded by Myh2, Myh1, and Myh4, respectively [10]. The distribution of different MHCs and fibre types varies in a muscle-specific as well as a species-specific manner [3, 15] and depending on the function, the anatomical location, and structure of the muscle [3, 12, 14]. For example, limb muscles predominantly contain type I fibres [3]. In the orofacial muscles, especially the masseter muscle, a different fibre distribution has been reported showing a wide variation in fibre type composition as demonstrated in biopsy studies [16]. Presumably depending on functional partitioning of activity of this muscle [6], a predominance of type I fibres in the anterior part and a general presence of hybrid fibres have been described as a normal feature of this muscle in humans [17, 18]. For temporal muscle a predominance of type I fibres in the anterior part (46%) [19] and lower portion of type I fibres (24%) in the posterior part may be also due to functional compartioning.

Muscle fibres are versatile and dynamic entities capable of adjusting their phenotypic properties under various conditions and in response to altered functional demands and display a great adaptive potential [8, 20]. The dynamic nature of skeletal muscle fibres is achieved by the adaption of its MyHCs composition [1, 21], and changes in its expression result in fibre type transitions [8]. This process can be regarded as a significant contribution to improve survival [8]. In developing muscles, fibre composition of individual muscle groups varies dramatically [12], fibre transitions is usually seen, and MHCs expression is regulated by neural, hormonal, mechanical, and other factors [9, 22, 23], but transitions of fibre types can also be found in adult muscle fibres due to biological ageing [2, 8, 24], activation intensity [2], neuromuscular activity [8, 25] or electrical stimulation [26, 27], hormone levels [2, 8, 28], exercises [29–32], training, during muscle atrophy induced by denervation [33], physical damage, and also muscle disease [34].

Duchenne muscular dystrophy (DMD), a X-linked recessive disease [35, 36], is one the most common and most serious myopathies which affects approximately 1 : 3500 life born males [36, 37]. Its onset is between 2 and 5 years of age [35] with clinical symptoms of muscle weakness in the limbs and pseudohypertrophy of calf muscles [38, 39]. At the age of 10–12 years, due to the loss of ambulation, the patients are wheelchair bound [40, 41] and usually the onset of masticatory involvement comes along with that time span. Interestingly the orofacial muscles are affected approximately 2 years later [42], which results in muscle imbalance and severe craniofacial and dental abnormalities [42–44].

The disease is caused by mutations in the dystrophin gene [35], encoding for the sarcolemmal protein product dystrophin, which is part of the DGC (dystrophin glycoprotein complex) and links the motor protein actin to the cytoskeleton [45]. It thus provides mechanical stability to striated muscles [46]. Absence of dystrophin leads to disintegration of the DGC, instability of muscle cell membrane, uncontrolled calcium influx [47], sterile inflammation, and progressive muscle degeneration [35, 48].

Currently the best-characterized and most widely used [49] animal model for DMD is the naturally occurring dystrophin-deficient mdx-mouse [50, 51], which carries a null mutation in exon 23 of the dystrophin gene [35, 47, 49]. Mdx-mice are a genetically biochemical homologue of the disease [37] but show a relative mild pathology [47], and severity of the phenotype is less than that in the human condition [36, 52]. Mdx-mice display hallmark symptoms of the diseases, changes in skeletal muscle histology, respiratory insufficiency, cardiomyopathy, and muscle weakness [36].

Botulinumtoxin A (BTX-A), a neurotoxin that blocks the release of acetylcholine at the neuromuscular junction and thus muscle contraction [53, 54] may lead to loss of muscle function caused by muscle atrophy [55, 56]. The therapeutic effects of BTX-A first appear in 1 to 3 days, peak in 1 to 4 weeks, and decline after 3 to 4 months [57].

Due to the fact that the mdx-mouse shows a higher regenerative capacity that ensures a more benign phenotype and essentially normal function [58], there is only a restricted time slot for research. To extend the time frame for evaluation of muscle influence on craniofacial growth and deformities, the purpose of this study was to determine the effects of BTX-A injection in the right masseter muscle of healthy and mdx-mice on the MyHC expression and protein content, to draw conclusions concerning remodelling and adaption of the injected muscle as well as the adjacent noninjected masseter, temporal, and tongue muscles.

## 2. Materials and Methods

In this study the experimental protocol and all procedures were approved by the Laboratory Animal Research Committee of Saxony with the number 24-9168.11-1/2013-46.

### 2.1. Animals

As subjects mice of the line C57Bl/10ScSn (control group, *n* = 10) and C57/Bl10ScSn-Dmdy (mdx) (test group, *n* = 10) of both genders, aged 100 days at baseline of the trial and a body weight of approximately 30 g, were used in this study. The mice originally obtained from Jackson Laboratory (Bar Harbor, USA) were borne in the laboratory animal experimental centre of Dresden and up to the beginning of the experiments housed in standard cages in groups of 2–6 animals under standardised conditions in a temperature-controlled room of 21–23°C maintained on a 12:12 h light-dark cycle with standard mice chow and water available ad libitum.

### 2.2. Chemodenervation Using Botulinumtoxin A (BTX-A)

For chemodenervation the mice were temporally anesthetized by an intraperitoneal injection consisting of a mixture of 10% ketamine (Ceva, Tiergesundheit GmbH, Düsseldorf, Germany) and 2% Rompun (Bayer, HealthCare AG, Leverkusen, Germany) in a relation to 3 : 2 at a dose of 0.1 mL per 100 g biomass.

To induce muscle paralysis, one single specific intramuscular injection of 0.025 mL BTX-A (Botox®, Allergan®, Irvine, California, USA; 1.25 IU/0.1 mL in physiologic NaCl-solution) was administered in the superficial and deep venter of the right masseter muscle. Both, healthy and mdx-mice were injected.

Postinjection care included a three-time daily control of the health state of the mice after they had awoken from anaesthesia and the verification of paralysis. The effects of chemodenervation after BTX-A injection usually start delayed. Paralysis in the BTX-A injection masseter is typically evident by the refusal of solid food and tooth chattering 3 days after injection. Due to that fact, the mice were kept under standard conditions described above with the possibility to choose between soft or solid food for the first 7 days. After that time, only solid food was offered. Until the terminal experiments the weight of the mice was controlled regularly.

### 2.3. Muscle Sample Preparation and Processing

21 days after intramuscular BTX-A injection, the animals were painlessly killed accordingly of the international guidelines for animal protection by means of an overdose of Isoflurane. The head was immediately separated from the body and the following muscles were carefully dissected and harvested by the same trained operator: masseter muscle (superficial venter) and temporal muscle from the injection side and contralateral side, respectively, as well as the tongue muscle.

According to Baverstock et al. [59], the dissected masseter muscle tissue corresponded to the superficial and (in parts) the deep masseter, accounting for 19% and 33% of overall masticatory muscle mass, respectively. The dissected temporal muscle, usually described as consisting of two parts and in the mouse distinguished as lateral and medial part, corresponded to the medial temporal muscle, which accounts for 77% of overall temporal muscle mass and is the larger portion of both parts [59].

For each of the examined muscles mRNA expression and protein content of myosin heavy chain (MyHC) isoforms were assessed. In addition, directly after dissection the right and left masseter muscles were weighted with a precision balance for direct comparison of muscle wet mass of the injected as well as the untreated contralateral side. The muscles were then flash-frozen in liquid nitrogen at −173°C and up to subsequent analysis stored in a deep fridge at −80°C.

### 2.4. RNA-Extraction and Reverse Transcription Reaction

Total RNA isolation from the muscle tissues was performed using Trizol (QIAzol Lysis Reagent QIAGEN, Hilden, Germany) and the RNeasy® Mini Kit (QIAGEN, Hilden, Germany) according to manufacturer's instructions. RNA concentrations and purity of the eluate were determined photometrically by ultraviolet absorbance measurements in the Eppendorf BioPhotometer® (Eppendorf Vertrieb Deutschland GmbH, Wesseling-Berzdorf, Germany) at a wavelength of 260 nm, a background compensation for the absorbance at 280 nm, and distilled water for calibration. Reverse transcription for synthesis of cDNA was performed in the Thermocycler TOptical (Analytik Jena AG, Jena, Germany) using an amount of 200 ng RNA and the innuSCRIPT Reverse Transcriptase in Nucleotide Mix and Random primer (Analytik Jena AG) following manufacturer's protocol.

### 2.5. qRT-PCR (Quantitative Real-Time Polymerase Chain Reaction)

The mRNA quantification of four different myosin heavy chain isoforms (Myh1, Myh2, Myh4, and Myh7) in the concerning muscle tissues was performed by qRT-PCR (Taq-Man® Assays; PE Applied Biosystems®, Weiterstadt, Germany) in comparison to 18S rRNA (Eucaryotic 18S rRNA Endogenous control: 4310893E; PE Applied Biosystems®, Weiterstadt, Germany) using gene-specific TaqMan PCR probes and primers which have been previously described elsewhere [60]. The master mix contained innuMix qPCR Master Mix Probe (Analytik Jena AG), 10x specific probes and primers, and RNase free water. For quantification of gene expression of the different MyHCs, 8 ng cDNA was used in a final volume of 20 *μ*L. Gene amplification was performed with the TOptical cycler (Analytik Jena AG) at 95°C for 10 minutes for initial denaturation followed by 40 cycles, in each case 10 seconds at 95°C and 45 seconds at 60°C. Absolute copy numbers of the studied genes and 18S cDNA were determined using calibration curves generated with cloned PCR fragment standards [60]. Copy numbers of individual transcripts were given in relation to those of 18S cDNA. Each probe was performed twice and a “nontemplate control” was carried out parallel in all experiments to validate results.

### 2.6. Western Blot

To extract muscle protein, the interphase and phenophase from the RNA isolation protocol were used. Protein isolation was performed following the manufacturer's protocol (isolation of DNA and protein from QIAzol Reagent-lysed samples (RY16 May-04) (QIAGEN, Hilden, Germany)). To identify the MyHC isoforms, 15 *μ*g of proteins from each muscle was loaded onto Citerion™ TGX Stain-free™ Precast Gels (Bio-RAD Laboratories GmbH, Munich, Germany) and separated by 100 V (constant Voltage) for 60 minutes. Following the electrophoresis, the proteins were transferred to the Western blot membrane, using the Trans-Blot® Semi-Dry transfer system (Trans-Blot® Turbo™ Midi PVDF Transfer Packs; Bio-RAD Laboratories GmbH) and the Trans-Blot Turbo blotting apparatus (Bio-RAD Laboratories GmbH). After protein transfer to the PVDF membrane, membranes were blocked over night at 4°C with 5% dry milk in phosphate-buffered saline (PBS) buffer with Tween.

Blots were incubated with monoclonal antibodies against MyHC proteins (anti-fast skeletal myosin antibody [My-32] (1 : 1000; Abcam, Cambridge, UK) and monoclonal Anti-Myosin (Skeletal, Slow) antibody produced in mouse (clone NOQ 7.5.4D; 1 : 1000; Sigma-Aldrich GmbH, Munich, Germany) diluted in PBS containing 5% dry milk and 0.025% NaN_3_ for 2-3 h at room temperature. Horseradish peroxidase- (HRP-) conjugate goat anti-mouse immunoglobulins (1 : 5000; Dako, Hamburg, Germany) were used afterwards. Bound antibodies were detected and visualized using an enhanced chemiluminescence system (WesternBright Chemilumineszenz Substrat Quantum, Advansta Inc., Menlo Park, USA). On each gel monoclonal anti-glyceraldehyde-phosphate dehydrogenase (GAPDH) antibody (clone 6C5; 1 : 1000; Millpore, Billerica, Massachusetts, USA) severed as loading control of the gels to finally calculate the protein content (incubation at room temperature for 2 h). Quantitative analyses of protein bands from MyHC isoforms and GAPDH in the examined masticatory muscle of the 100-day-old healthy and mdx-mice were undertaken using GelScan 5.2 software (Serva, Heidelberg, Germany). Mean optical density ± SEM are given in all cases for *n* = 4 muscle samples of different animals and three-independent Western blot analysis.

### 2.7. Statistics

To evaluate differences in the mRNA expression and protein content of the different fibre types in the analysed masticatory muscles of healthy and mdx-mice, statistical analysis was performed using the Software SigmaStat® Version 3.5 (Systat Software Inc., San Jose, California, USA) and Mann Whitney* U* test. In case of a normal distribution, the* t*-test was applied. Results *p* ≤ 0.05 were regarded as statistically significant.

## 3. Results

### 3.1. Body Weight and Muscle Dimensions

An initial decrease in body weight in the very early postinjection days, which might be attributed to the beginning paralysis and reduced food ingestion, was followed by a steady increase during the experimental protocol up to its closure. A statistical difference in body weight between the control and mdx group was not obvious at any time. During the entire experimental setup the body weight was similar in both groups.

**Figure 1:**
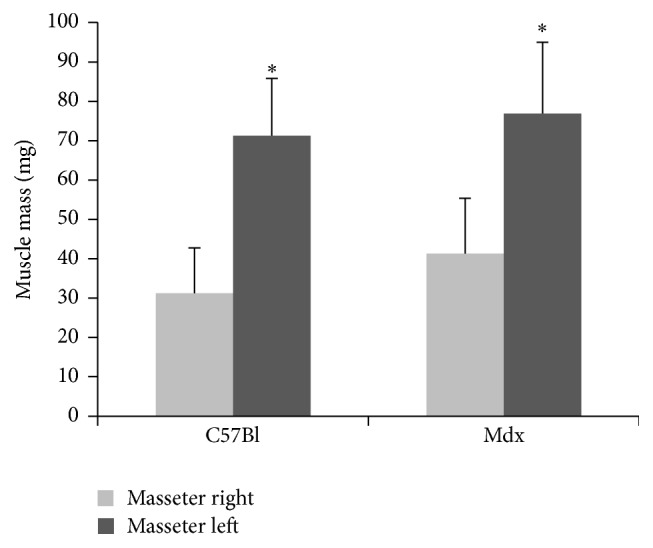
Muscle weight of treated (right) and untreated (left) masseter in control (C57Bl; *n* = 10) and mdx-mice (*n* = 10) 21 days after BTX-A injection. Mean values ± standard deviations; Student's* t*-test (^*∗*^ *p* ≤ 0.05).

To verify BTX-A induced atrophy, muscle mass of the injected masseter muscle and the corresponding contralateral noninjected muscle was measured. A comparison of the muscle tissue mass revealed a statistically significant difference between the weight and size of the injected (right) and untreated (left) masseter muscles for both control and mdx-mice (Figure 1). Compared to the untreated left masseter muscles, injected right masseter muscles were 56% and 45% smaller in the control and mdx group, respectively.

### 3.2. mRNA Expression of MyHC Isoforms

At first, the already known expression difference between dystrophic and healthy mice was also detectable, such as a decrease of Myh2 mRNA and an increase of Myh4 mRNA in dystrophic masseter muscle as well as an increase of Myh7 mRNA expression levels (encoding for fibre type I) in dystrophic temporal muscle compared to control.

21 days after BTX-A injection in the right masseter muscle, mRNA expression of MyHCs in the extracted muscle tissues of injected atrophic masseter muscle of control-mice differed significantly from the untreated noninjection side. A significant decrease of Myh4 mRNA and Myh1 mRNA encoding for the fast fibre types IIb and IIx/d, respectively, could be detected, whereas Myh2 and Myh7 mRNA expression, corresponding to the fibre types IIa and I, were significantly increased. In control temporal muscle tissue of healthy mice no differences between the right and left side were found. In tongue muscle a high expression level of Myh4 mRNA in the control as well as mdx group, with no statistically significant differences between both groups, could be detected.

The mRNA expression of all tested MyHC isoforms remained unchanged in mdx muscle tissue from both sides. No differences between BTX-A treated and untreated masseter muscle as well as right and left side could be observed in this mice strain (Table 1).

### 3.3. Protein Content

**Figure 2:**
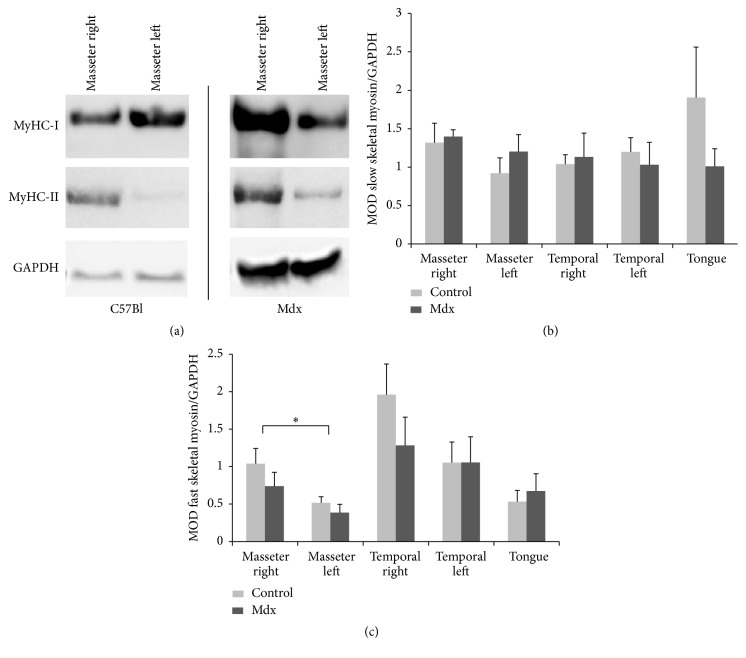
Western blot analysis of fast and slow skeletal muscle myosin extracted from 100-day-old mice (control/mdx) 21 days after BTX-A injection into the right masseter muscle. (a) Representative blots, (b) quantitative analyses of slow myosin (MyHC-I), and (c) fast myosin (MyHC-II) compared to GAPDH in masticatory muscle of BTX-A treated control- (C57Bl) and mdx-mice. Mean optical density (MOD) ± SEM are given in all cases for *n* = 4 muscle samples of different animals and three-independent Western blot analysis (Student's* t*-test; ^*∗*^ *p* ≤ 0.05 treated versus untreated masseter muscle).

Semiquantitative analysis of fast and slow skeletal myosin proteins were performed with Western blot analysis. Single immune-reactive bands of approximately 220 kDa were detected. These bands correspond to the expected molecular weights of slow as well as fast myosin, respectively (Figure 2(a)). The quantitative evaluation of Western blots only showed significant increased protein levels of MyHC-II in BTX-A treated control masseter muscle compared to contralateral untreated masseter muscle tissue of control-mice (*p* = 0.006; Figure 2(c)). In neither other muscle samples from control-mice nor all muscle samples from dystrophic mice differences in the expression levels of fast and slow myosin heavy chains could be detected (Figure 2).

## 4. Discussion

In the present study the effects of different masticatory muscles after BTX-A injection in the right masseter of healthy and mdx-mice muscles were evaluated concerning mRNA and protein expression of MyHC isoforms. BTX-A was used to reduce masticatory function and to prolong dystrophic features of muscle fibres in the masseter muscle of mdx-mice. Usually muscle pathology of mdx-mice is most pronounced and peaks at the age of 3-4 weeks [35, 61] and goes to cycles of de- and regeneration by 9–12 weeks of age, where regeneration overcomes degeneration of muscle fibres [62, 63]. In our study 100-day-old control and dystrophic mice were used. It has previously been shown that muscle fibres of mdx-mice of this age have centralized nuclei in 75% of all muscle fibres, a typical sign of muscle fibre regeneration [64].

BTX-A injection and inactivation of the right masseter muscle clearly resulted in a reduction of muscle weight 21 days after injection. It is well known that BTX-A treatment indirectly induces masticatory hypofunction and muscle atrophy due to chemoinactivation [53]. Changes in muscle weight after BTX-A injection could also previously be found [65] and marked atrophy of paralyzed muscle fibres [5, 55] is a common feature also seen under clinical conditions taking advantage of BTX-A treatment for masseter hypertrophy [66, 67] and bruxism [68, 69]. In animal experiments, the muscle fibres begin to show atrophy histologically within 10–14 days after injection and this atrophy continues to develop over 4–6 weeks [70]. As shown previously, BTX-A influences the expression of MyHC isoforms. After BTX-A injection into the right masseter muscle of pigs a fibre shift towards faster isoforms in the injected as well as to slower isoforms in the noninjected masseter muscle could be demonstrated [71]. Slow-to-fast fibre shift is also well known from other denervation studies where no nerve stimulation exists [5]. In our study at protein level similar effects could be seen in injected control masseter muscle with a statistically significant increase of type II myofibres compared to the contralateral noninjection side, whereas treated dystrophic masseter muscle does not show any statistically significant differences compared to untreated masseter muscle of mdx-mice.

By contrast, at mRNA level some other effects have been demonstrated in wild-type mice. In the right masseter muscle of control-mice a decrease in MyHC-IIb and -IIx mRNA expression as well as an increase in MyHC-IIa encoding for the slowest form of type II myofibres and an increase of MyHC-I encoding for slow muscle fibres could be identified. It is well known that healthy mouse masseter muscle (superficial part) predominantly consists of fast type IIb myofibres [72, 73]. A reduction of fast muscle fibres is typically seen in dystrophic muscles. Petrof et al. have proved preferential degeneration of type II fibres in mdx limb and trunk muscles [74] and in dystrophic masticatory muscles reduced MyHC-IIb mRNA expression was also detected [60]. Furthermore, it was reported that the IIb fibres degenerate first in DMD patients [75]. Analyses of MHC isoforms in the affected diaphragm of canine X-linked muscular dystrophy (CXMDJ) also indicated a marked increase of type I and decrease of type IIa myosin isoforms [76]. In histological studies a fibre shift with more pronounced portion of slow type I fibres has been described for the superficial masseter muscle as a hallmark of disease progression obvious in 14-week-old mdx-mice [77]. Lee et al. by their study indicated that after degeneration the regenerated muscle acquires muscle fibre characteristics entirely different from those in its normal counterpart, with a very strong expression of MyHC-I in mdx-mouse masseter at 9 weeks of age [63]. The MyHC-IIx isoform has already been reported to represent the transition from fast type II to slow type I myofibres [22] and subsequently this transition results in a decrease of type II fibres. Based on these findings, it is to be assumed that chemical denervation, induced by BTX-A, simulates dystrophic appearance in healthy mice masticatory muscles with regard to fibre composition.

Muscles can also induce a fibre shift towards slower isoforms to adapt to varying functional load and endurance training [78]. In 10-week-old pigs, after chronic sagittal advancement of the mandible, a significant increase in the cross-sectional area of type I fibres and type I MyHC expression in the anterior part of the masseter, distal part of the temporal and the medial pterygoid muscle could be shown, which was simultaneously accompanied by a comparable decrease in type II MyHCs in these muscles [21]. Step-by-step transition of muscle fibres from type IIb via type IIa to type I under activity was also found by Goldspink [79] and Pette and Staront [80] and they stated that under higher muscle stress the increase in cross-sectional area of type I fibres is more efficient for long-term energy accumulation. In our study, in healthy mice after BTX-A injection a decrease in fast muscle fibres was observed in the untreated masseter muscle, which was statistically significantly different from the injection side showing at once an increase in type II fibres. It is well known that stretch overload influences fibre type transition towards slower MyHC isoform expression [8], and the same transition takes place in case of functional overload on skeletal muscles [81]. In the right temporal muscle of control-mice an increase of MyHC-II protein expression could also be observed, but these results were not statistically significantly different from the contralateral side. However this tendency of type II fibre increase seen could possibly be traced back to a compensatory effect of functional loss of the right masseter muscle and the rapid force increase and high stress, which is known to induce a fibre shift towards faster isoforms [78]. Harzer et al. emphasized that short-time and long-term strain on muscles due to endurance and fast-force training lead to different reactions, respectively. Though endurance training promotes a fibre shift from fast type II muscle fibres to slow type I with a more efficient energy supply during constant load [82], whereas fast-force training promotes a fibre shift in the opposite direction [78]. It can be assumed that due to the BTX-A induced inactivation of the right masseter muscle, by simultaneous preservation of the chewing function, other muscle groups might have experienced more stress and might have adapted by changing their fibre type composition. Nevertheless this effect was only significant for the left masseter muscle in control-mice. On the other hand the effect seen at protein level in healthy mice could also be explained by BTX-A induced fibre shift towards faster isoforms in the injected muscle tissue whereas fibre type composition of left masseter muscle might not have been changed. This explanation seems to be more suitable because at protein level a statistically significant increase of type I fibres in left masseter muscle tissue was missing.

In contrast to healthy control-mice, muscles of mdx-mice showed no remarkable changes induced by BTX-A injection. It had been expected that the dystrophic injected masseter muscle had been reacted in the same way the healthy muscle did, but at protein level a statistically significant shift towards faster isoforms could not be seen in this muscle although such a tendency could be found. One explanation could be that the regeneration potential of this muscle was already exhausted due to the disease, and a shift towards faster isoforms could not be realized. Another possible explanation is due the role of dystrophin, which attributes particular importance in the expression of type IIb fibres. Webster et al. already emphasized that fast fibres are preferentially affected in DMD, and it has been suggested that dystrophin gene product plays a specific and essential role in IIb fibre function, a subpopulation of muscle fibres specialized to respond to the highest frequency of neuronal stimulation with maximal rates of contraction [75]. Possibly dystrophin is essential for accepting the high-frequency activity of fast motor neurons to carry out the high-frequency contraction demanded for type IIb muscle fibres [75]. In this case, the defect in the muscle fibre would be manifested only under neural influence [83]. Hence, with innervation being absent no degeneration of IIb fibres should occur. Vice versa, even though with innervation and strong IIb mRNA expression being present, which could be seen on mRNA level in our study, on account of dystrophin deficiency, no translation for MyHC-IIb exists. So that the last mentioned explanation is more appropriate to explain our results. Perhaps by this reason the reaction to BTX-A injection occurs temporally delayed in this mouse strain. Usually mRNA expression goes ahead to protein expression which occurs as time-delayed. In this context the fact that only one time point was investigated needs to be considered critically. On the other hand, it was of special interest to investigate both protein and mRNA expression in the same muscle tissues after BTX-A injection to estimate changes occurring at exactly one time point during the process of muscle fibre adaption. The mRNA response to stress is very rapid; thus mRNA content represents a steady state of muscle adaption, but with the additional analysis of protein content more distinctive effects of BTX-A injection could be demonstrated.

Effects on mRNA expression encoding for MyHC isoforms found in injected muscles in our study, especially in healthy mice, could also be attributed to reinnervation process which is known to occur 3-4 weeks after denervation [84]. Nayyar et al. stated that after that time, at the cellular level, upregulation of the muscle nicotinic receptors and accordingly reappearance of muscle innervation, due to sprouting could be found in mice after a single BTX-A injection [84]. Hence, the results presented here may reflect the sum of fibre shifts in specific masticatory muscles evoked by BTX-A induced immobilisation and subsequent recovery of muscle function, reflected by fibre type diversity in different muscles and accumulation of specific types during fibre shift to one end of the fibre spectrum.

In conclusion, our study confirmed that a fibre shift due to BTX-A injection is possible in healthy mice muscle tissue, inducing different fibre type transformations. Likewise, the mRNA MyHC expression demonstrated similar changes to those observed in the untreated dystrophic muscles. However, in dystrophic mice BTX-A injection did elicit neither detectable direct or indirect compensatory fibre shifts, nor a prolongation of the dystrophic phenotype. Further research at different time points is necessary to fully elucidate the impact of BTX-A injection on masticatory muscles and to better estimate whether this medication or treatment method is likely to be able to selectively switch off the active regeneration processes of the musculature in the mdx-mouse and therefore to be able to use this effect for research concerning craniofacial changes induced by functional adaptions.

## Figures and Tables

**Table 1:** mRNA expression of MyHC isoforms (Myh1, Myh2, Myh4, and Myh7) in BTX-A treated and untreated muscle tissue, 21 days after injection into the right masseter muscle of 100-day-old mice (control/mdx; *n* = 10 each). Means ± standard deviations; Student's *t*-test  ^*∗*^ *p* ≤ 0.05 treated versus untreated masseter muscle;  ^#^ *p* ≤ 0.05 control versus mdx. Significant values are indicated by bold lettering.

Gene name	Fibre type	Masseter right		Masseter left		Temporal right		Temporal left		Tongue	
		Control	Mdx	Control	Mdx	Control	Mdx	Control	Mdx	Control	Mdx
Myh 1	IIx/d	***1.06* ± *0.71***	1.37 ± 0.64	***1.65 ± 0.87***	1.19 ± 0.56	1.27 ± 0.88	1.14 ± 0.67	1.68 ± 1.35	1.82 ± 1.2	0.25 ± 0.16	0.63 ± 0.67
		^*∗*^ *p* = 0.041									

Myh 2	IIa	***2.29 ± 0.79***	***1.23 ± 0.61***	***0.77 ± 0.59***	0.75 ± 0.45	4.41 ± 2.97	3.41 ± 0.54	3.67 ± 1.85	4.37 ± 3.05	0.02 ± 0.02	0.07 ± 0.09
		^*∗*^ *p* < 0.001	^#^ *p* = 0.014								

Myh 4	IIb	***1.18 ± 0.90***	***2.63 ± 1.57***	***2.99 ± 2.43***	2.60 ± 1.85	9.36 ± 6.66	9.80 ± 8.79	19.47 ± 17.15	10.12 ± 12.48	25.8 ± 17.71	33.02 ± 17.29
		^*∗*^ *p* = 0.023	^#^ *p* = 0.019								

Myh 7	I	**0.000133 ± 3.15E5**	0.000084 ± 3.53*E*5	**0.000053 ± 8.98E6**	0.000041 ± 8.59*E*6	0.000151 ± 2.93*E*5	0.000215 ± 3.77*E*5	**0.000113 ± 1.8E5**	**0.000261 ± 7.27E5**	0.000005 ± 2.36*E*6	0.00001 ± 6.07*E*6
		^*∗*^ *p* = 0.012						^#^ *p* = 0.019			
